# Supplementary material for: Modeling glioblastoma heterogeneity as a dynamic network of cell states
Source: Mol Syst Biol. 2021 Sep 16;17(9):e10105. doi: 10.15252/msb.202010105 (PMC8444284; doi:10.15252/msb.202010105)
Supplement: Supplementary file 6 — Source Data for Figure 5 [file MSB-17-e10105-s004.zip › Figure5A_sourcedata/GSEA_3017/nosigdb_stateB.GseaPreranked.1622795021725/RICHARDS_NOWAKOWSKI_SCIENCE_2017_MGE_IPC1_UPREG.html]

Details for gene set RICHARDS\_NOWAKOWSKI\_SCIENCE\_2017\_MGE\_IPC1\_UPREG[GSEA]

|  || Dataset | state43017 |
| Phenotype | NoPhenotypeAvailable |
| Upregulated in class | na\_pos |
| GeneSet | RICHARDS\_NOWAKOWSKI\_SCIENCE\_2017\_MGE\_IPC1\_UPREG |
| Enrichment Score (ES) | 0.54656786 |
| Normalized Enrichment Score (NES) | 3.4559264 |
| Nominal p-value | 0.0 |
| FDR q-value | 0.0 |
| FWER p-Value | 0.0 |
Table: GSEA Results Summary

  

Fig 1: Enrichment plot: RICHARDS\_NOWAKOWSKI\_SCIENCE\_2017\_MGE\_IPC1\_UPREG      
 Profile of the Running ES Score & Positions of GeneSet Members on the Rank Ordered List

  

| PROBE | GENE SYMBOL | GENE\_TITLE | RANK IN GENE LIST | RANK METRIC SCORE | RUNNING ES | CORE ENRICHMENT || 1 | HIST1H4C |  |  | 0 | 1.373 | 0.0125 | Yes |
| 2 | HIST1H1B |  |  | 1 | 1.272 | 0.0242 | Yes |
| 3 | HIST1H1D |  |  | 2 | 1.180 | 0.0349 | Yes |
| 4 | TOP2A |  |  | 3 | 1.079 | 0.0448 | Yes |
| 5 | HIST1H1C |  |  | 4 | 1.041 | 0.0543 | Yes |
| 6 | HIST1H1E |  |  | 5 | 0.983 | 0.0633 | Yes |
| 7 | RRM2 |  |  | 7 | 0.875 | 0.0694 | Yes |
| 8 | FAM111A |  |  | 10 | 0.813 | 0.0730 | Yes |
| 9 | UBE2C |  |  | 11 | 0.802 | 0.0803 | Yes |
| 10 | TPX2 |  |  | 12 | 0.801 | 0.0876 | Yes |
| 11 | NUSAP1 |  |  | 13 | 0.801 | 0.0949 | Yes |
| 12 | HMGB2 |  |  | 14 | 0.786 | 0.1021 | Yes |
| 13 | FBXO5 |  |  | 15 | 0.785 | 0.1093 | Yes |
| 14 | NCAPG |  |  | 16 | 0.778 | 0.1164 | Yes |
| 15 | SPC25 |  |  | 17 | 0.763 | 0.1234 | Yes |
| 16 | PRC1 |  |  | 18 | 0.756 | 0.1303 | Yes |
| 17 | CDCA2 |  |  | 19 | 0.751 | 0.1371 | Yes |
| 18 | ESCO2 |  |  | 20 | 0.742 | 0.1439 | Yes |
| 19 | TACC3 |  |  | 21 | 0.741 | 0.1507 | Yes |
| 20 | GTSE1 |  |  | 22 | 0.732 | 0.1574 | Yes |
| 21 | HJURP |  |  | 23 | 0.731 | 0.1640 | Yes |
| 22 | KIF23 |  |  | 24 | 0.721 | 0.1706 | Yes |
| 23 | ASPM |  |  | 26 | 0.695 | 0.1751 | Yes |
| 24 | NDC80 |  |  | 27 | 0.695 | 0.1814 | Yes |
| 25 | ATAD2 |  |  | 28 | 0.685 | 0.1877 | Yes |
| 26 | KIF15 |  |  | 29 | 0.684 | 0.1939 | Yes |
| 27 | ARHGAP11A |  |  | 30 | 0.683 | 0.2002 | Yes |
| 28 | KPNA2 |  |  | 33 | 0.676 | 0.2025 | Yes |
| 29 | CENPF |  |  | 34 | 0.670 | 0.2086 | Yes |
| 30 | KIF4A |  |  | 37 | 0.662 | 0.2109 | Yes |
| 31 | ANLN |  |  | 39 | 0.659 | 0.2150 | Yes |
| 32 | RAD51AP1 |  |  | 40 | 0.657 | 0.2210 | Yes |
| 33 | SMC4 |  |  | 41 | 0.655 | 0.2270 | Yes |
| 34 | MELK |  |  | 42 | 0.655 | 0.2330 | Yes |
| 35 | CENPE |  |  | 43 | 0.654 | 0.2389 | Yes |
| 36 | NUF2 |  |  | 44 | 0.651 | 0.2449 | Yes |
| 37 | PRR11 |  |  | 45 | 0.649 | 0.2508 | Yes |
| 38 | DLGAP5 |  |  | 46 | 0.647 | 0.2567 | Yes |
| 39 | KIFC1 |  |  | 47 | 0.645 | 0.2626 | Yes |
| 40 | KIF18A |  |  | 48 | 0.639 | 0.2685 | Yes |
| 41 | NCAPG2 |  |  | 50 | 0.636 | 0.2724 | Yes |
| 42 | CENPA |  |  | 51 | 0.636 | 0.2782 | Yes |
| 43 | CDCA5 |  |  | 52 | 0.632 | 0.2839 | Yes |
| 44 | CKAP2 |  |  | 53 | 0.628 | 0.2897 | Yes |
| 45 | CCNA2 |  |  | 54 | 0.626 | 0.2954 | Yes |
| 46 | AURKA |  |  | 55 | 0.617 | 0.3010 | Yes |
| 47 | CKAP2L |  |  | 59 | 0.611 | 0.3009 | Yes |
| 48 | CENPU |  |  | 60 | 0.605 | 0.3064 | Yes |
| 49 | CENPK |  |  | 62 | 0.603 | 0.3100 | Yes |
| 50 | TYMS |  |  | 63 | 0.601 | 0.3155 | Yes |
| 51 | BUB1B |  |  | 64 | 0.596 | 0.3210 | Yes |
| 52 | CCDC34 |  |  | 65 | 0.595 | 0.3264 | Yes |
| 53 | KIF2C |  |  | 66 | 0.591 | 0.3318 | Yes |
| 54 | SMC2 |  |  | 68 | 0.586 | 0.3352 | Yes |
| 55 | CDK1 |  |  | 69 | 0.585 | 0.3406 | Yes |
| 56 | H2AFX |  |  | 71 | 0.576 | 0.3440 | Yes |
| 57 | CKS1B |  |  | 72 | 0.575 | 0.3492 | Yes |
| 58 | RACGAP1 |  |  | 75 | 0.572 | 0.3506 | Yes |
| 59 | FOXM1 |  |  | 76 | 0.570 | 0.3558 | Yes |
| 60 | MAD2L1 |  |  | 77 | 0.569 | 0.3610 | Yes |
| 61 | PLK1 |  |  | 78 | 0.567 | 0.3662 | Yes |
| 62 | DBF4 |  |  | 79 | 0.567 | 0.3714 | Yes |
| 63 | CDCA8 |  |  | 82 | 0.562 | 0.3727 | Yes |
| 64 | CKS2 |  |  | 83 | 0.558 | 0.3778 | Yes |
| 65 | ECT2 |  |  | 84 | 0.558 | 0.3829 | Yes |
| 66 | KIF14 |  |  | 85 | 0.557 | 0.3880 | Yes |
| 67 | UBE2S |  |  | 87 | 0.555 | 0.3912 | Yes |
| 68 | AURKB |  |  | 89 | 0.548 | 0.3943 | Yes |
| 69 | PIF1 |  |  | 90 | 0.545 | 0.3992 | Yes |
| 70 | SPC24 |  |  | 91 | 0.544 | 0.4042 | Yes |
| 71 | CCNB1 |  |  | 93 | 0.544 | 0.4073 | Yes |
| 72 | NCAPH |  |  | 94 | 0.543 | 0.4122 | Yes |
| 73 | PBK |  |  | 95 | 0.540 | 0.4172 | Yes |
| 74 | DEPDC1 |  |  | 97 | 0.536 | 0.4202 | Yes |
| 75 | SKA3 |  |  | 99 | 0.525 | 0.4230 | Yes |
| 76 | MXD3 |  |  | 104 | 0.519 | 0.4202 | Yes |
| 77 | CCNF |  |  | 106 | 0.513 | 0.4230 | Yes |
| 78 | CDKN3 |  |  | 108 | 0.513 | 0.4258 | Yes |
| 79 | CEP135 |  |  | 109 | 0.513 | 0.4304 | Yes |
| 80 | UBE2T |  |  | 110 | 0.512 | 0.4351 | Yes |
| 81 | CDCA3 |  |  | 111 | 0.511 | 0.4398 | Yes |
| 82 | DIAPH3 |  |  | 112 | 0.510 | 0.4444 | Yes |
| 83 | BUB1 |  |  | 113 | 0.508 | 0.4491 | Yes |
| 84 | TUBB4B |  |  | 114 | 0.507 | 0.4537 | Yes |
| 85 | RAD21 |  |  | 115 | 0.506 | 0.4583 | Yes |
| 86 | BIRC5 |  |  | 116 | 0.504 | 0.4629 | Yes |
| 87 | DTYMK |  |  | 118 | 0.503 | 0.4656 | Yes |
| 88 | CEP152 |  |  | 119 | 0.502 | 0.4702 | Yes |
| 89 | KIF18B |  |  | 123 | 0.494 | 0.4690 | Yes |
| 90 | SPDL1 |  |  | 126 | 0.492 | 0.4697 | Yes |
| 91 | TMPO |  |  | 127 | 0.492 | 0.4742 | Yes |
| 92 | ITGB3BP |  |  | 128 | 0.490 | 0.4787 | Yes |
| 93 | LMNB2 |  |  | 129 | 0.488 | 0.4831 | Yes |
| 94 | SKA1 |  |  | 131 | 0.487 | 0.4857 | Yes |
| 95 | SHCBP1 |  |  | 133 | 0.484 | 0.4882 | Yes |
| 96 | GMNN |  |  | 135 | 0.484 | 0.4907 | Yes |
| 97 | NCAPD2 |  |  | 136 | 0.483 | 0.4951 | Yes |
| 98 | TTK |  |  | 137 | 0.482 | 0.4995 | Yes |
| 99 | CENPL |  |  | 139 | 0.478 | 0.5020 | Yes |
| 100 | INCENP |  |  | 140 | 0.476 | 0.5063 | Yes |
| 101 | C21orf58 |  |  | 142 | 0.470 | 0.5087 | Yes |
| 102 | FANCD2 |  |  | 149 | 0.461 | 0.5015 | Yes |
| 103 | ORC6 |  |  | 150 | 0.461 | 0.5057 | Yes |
| 104 | KIF22 |  |  | 152 | 0.459 | 0.5080 | Yes |
| 105 | KNSTRN |  |  | 155 | 0.456 | 0.5084 | Yes |
| 106 | SPAG5 |  |  | 156 | 0.455 | 0.5125 | Yes |
| 107 | POLQ |  |  | 162 | 0.450 | 0.5071 | Yes |
| 108 | CDKN2C |  |  | 164 | 0.445 | 0.5093 | Yes |
| 109 | CDK5RAP2 |  |  | 173 | 0.438 | 0.4981 | Yes |
| 110 | BRCA2 |  |  | 174 | 0.436 | 0.5021 | Yes |
| 111 | DEPDC1B |  |  | 177 | 0.436 | 0.5023 | Yes |
| 112 | MKI67 |  |  | 179 | 0.436 | 0.5043 | Yes |
| 113 | RHNO1 |  |  | 183 | 0.432 | 0.5026 | Yes |
| 114 | CDC20 |  |  | 184 | 0.431 | 0.5065 | Yes |
| 115 | USP1 |  |  | 185 | 0.430 | 0.5104 | Yes |
| 116 | MIS18BP1 |  |  | 186 | 0.430 | 0.5144 | Yes |
| 117 | MGME1 |  |  | 188 | 0.428 | 0.5164 | Yes |
| 118 | BARD1 |  |  | 189 | 0.427 | 0.5203 | Yes |
| 119 | CDKN2D |  |  | 190 | 0.427 | 0.5242 | Yes |
| 120 | HMMR |  |  | 191 | 0.426 | 0.5281 | Yes |
| 121 | CKAP5 |  |  | 192 | 0.426 | 0.5320 | Yes |
| 122 | MND1 |  |  | 193 | 0.424 | 0.5358 | Yes |
| 123 | NEK2 |  |  | 194 | 0.423 | 0.5397 | Yes |
| 124 | MIS18A |  |  | 196 | 0.421 | 0.5416 | Yes |
| 125 | CXXC5 |  |  | 210 | 0.408 | 0.5206 | Yes |
| 126 | ZWINT |  |  | 212 | 0.407 | 0.5225 | Yes |
| 127 | H2AFZ |  |  | 216 | 0.405 | 0.5205 | Yes |
| 128 | MNS1 |  |  | 219 | 0.401 | 0.5203 | Yes |
| 129 | UTRN |  |  | 220 | 0.400 | 0.5240 | Yes |
| 130 | CENPO |  |  | 223 | 0.398 | 0.5238 | Yes |
| 131 | STIL |  |  | 231 | 0.393 | 0.5141 | Yes |
| 132 | PCM1 |  |  | 232 | 0.393 | 0.5177 | Yes |
| 133 | EZH2 |  |  | 236 | 0.388 | 0.5155 | Yes |
| 134 | VRK1 |  |  | 238 | 0.388 | 0.5171 | Yes |
| 135 | PARPBP |  |  | 240 | 0.387 | 0.5188 | Yes |
| 136 | HMGN2 |  |  | 243 | 0.386 | 0.5185 | Yes |
| 137 | SUGP2 |  |  | 244 | 0.385 | 0.5220 | Yes |
| 138 | RTKN2 |  |  | 248 | 0.379 | 0.5198 | Yes |
| 139 | CCNB2 |  |  | 250 | 0.379 | 0.5213 | Yes |
| 140 | JADE1 |  |  | 251 | 0.378 | 0.5248 | Yes |
| 141 | MPHOSPH9 |  |  | 254 | 0.375 | 0.5244 | Yes |
| 142 | PLK4 |  |  | 255 | 0.374 | 0.5278 | Yes |
| 143 | C18orf54 |  |  | 259 | 0.371 | 0.5255 | Yes |
| 144 | CENPJ |  |  | 261 | 0.370 | 0.5270 | Yes |
| 145 | GAS2L3 |  |  | 262 | 0.369 | 0.5304 | Yes |
| 146 | TUBB |  |  | 263 | 0.368 | 0.5337 | Yes |
| 147 | ASRGL1 |  |  | 266 | 0.366 | 0.5333 | Yes |
| 148 | CENPW |  |  | 267 | 0.366 | 0.5366 | Yes |
| 149 | TUBG1 |  |  | 268 | 0.365 | 0.5399 | Yes |
| 150 | CEP57L1 |  |  | 269 | 0.364 | 0.5433 | Yes |
| 151 | TUBA1C |  |  | 270 | 0.362 | 0.5466 | Yes |
| 152 | ANP32E |  |  | 280 | 0.358 | 0.5327 | No |
| 153 | CDC25B |  |  | 286 | 0.356 | 0.5265 | No |
| 154 | CALM2 |  |  | 290 | 0.354 | 0.5240 | No |
| 155 | HAUS8 |  |  | 293 | 0.353 | 0.5234 | No |
| 156 | NEIL3 |  |  | 304 | 0.345 | 0.5076 | No |
| 157 | CDC25C |  |  | 307 | 0.343 | 0.5069 | No |
| 158 | CENPN |  |  | 309 | 0.343 | 0.5081 | No |
| 159 | PKP4 |  |  | 311 | 0.342 | 0.5094 | No |
| 160 | RANGAP1 |  |  | 312 | 0.342 | 0.5125 | No |
| 161 | DEK |  |  | 316 | 0.341 | 0.5099 | No |
| 162 | PHF19 |  |  | 319 | 0.339 | 0.5092 | No |
| 163 | CIT |  |  | 326 | 0.336 | 0.5009 | No |
| 164 | NES |  |  | 334 | 0.332 | 0.4906 | No |
| 165 | BTG3 |  |  | 337 | 0.331 | 0.4898 | No |
| 166 | PHGDH |  |  | 344 | 0.328 | 0.4814 | No |
| 167 | ARHGAP11B |  |  | 356 | 0.323 | 0.4634 | No |
| 168 | LMNB1 |  |  | 363 | 0.321 | 0.4549 | No |
| 169 | PNRC2 |  |  | 364 | 0.321 | 0.4579 | No |
| 170 | XPO1 |  |  | 365 | 0.321 | 0.4608 | No |
| 171 | RBBP7 |  |  | 367 | 0.321 | 0.4618 | No |
| 172 | MID1 |  |  | 375 | 0.317 | 0.4514 | No |
| 173 | KIF20A |  |  | 392 | 0.311 | 0.4238 | No |
| 174 | CEP192 |  |  | 395 | 0.310 | 0.4229 | No |
| 175 | RHEB |  |  | 404 | 0.307 | 0.4105 | No |
| 176 | CENPC |  |  | 409 | 0.304 | 0.4056 | No |
| 177 | LRR1 |  |  | 410 | 0.304 | 0.4084 | No |
| 178 | LIN54 |  |  | 411 | 0.303 | 0.4112 | No |
| 179 | PTTG1 |  |  | 413 | 0.302 | 0.4120 | No |
| 180 | AHI1 |  |  | 425 | 0.299 | 0.3938 | No |
| 181 | ZMYM1 |  |  | 429 | 0.298 | 0.3909 | No |
| 182 | NUP107 |  |  | 430 | 0.298 | 0.3936 | No |
| 183 | G2E3 |  |  | 446 | 0.294 | 0.3677 | No |
| 184 | IQGAP3 |  |  | 447 | 0.294 | 0.3704 | No |
| 185 | TEX30 |  |  | 448 | 0.294 | 0.3731 | No |
| 186 | CCDC150 |  |  | 449 | 0.293 | 0.3758 | No |
| 187 | TERF1 |  |  | 451 | 0.293 | 0.3766 | No |
| 188 | MZT1 |  |  | 456 | 0.291 | 0.3716 | No |
| 189 | CBX5 |  |  | 457 | 0.291 | 0.3743 | No |
| 190 | SAE1 |  |  | 463 | 0.289 | 0.3674 | No |
| 191 | RFWD3 |  |  | 464 | 0.289 | 0.3700 | No |
| 192 | NUP50 |  |  | 470 | 0.287 | 0.3632 | No |
| 193 | KCTD9 |  |  | 471 | 0.287 | 0.3658 | No |
| 194 | ACTN1 |  |  | 475 | 0.286 | 0.3627 | No |
| 195 | NUCKS1 |  |  | 483 | 0.285 | 0.3520 | No |
| 196 | APOLD1 |  |  | 487 | 0.284 | 0.3489 | No |
| 197 | METTL4 |  |  | 488 | 0.283 | 0.3515 | No |
| 198 | CTDSPL2 |  |  | 495 | 0.282 | 0.3426 | No |
| 199 | IKBIP |  |  | 508 | 0.279 | 0.3224 | No |
| 200 | TNFAIP8L1 |  |  | 510 | 0.279 | 0.3230 | No |
| 201 | FAM83D |  |  | 511 | 0.279 | 0.3256 | No |
| 202 | TTF2 |  |  | 514 | 0.278 | 0.3243 | No |
| 203 | MBNL2 |  |  | 517 | 0.278 | 0.3230 | No |
| 204 | CDC27 |  |  | 520 | 0.278 | 0.3218 | No |
| 205 | SAP30 |  |  | 547 | 0.273 | 0.2748 | No |
| 206 | SCLT1 |  |  | 548 | 0.273 | 0.2773 | No |
| 207 | NIF3L1 |  |  | 561 | 0.270 | 0.2570 | No |
| 208 | BRD8 |  |  | 562 | 0.270 | 0.2594 | No |
| 209 | NUP205 |  |  | 571 | 0.269 | 0.2467 | No |
| 210 | CNTRL |  |  | 575 | 0.269 | 0.2434 | No |
| 211 | SAPCD2 |  |  | 578 | 0.268 | 0.2421 | No |
| 212 | CCDC18 |  |  | 586 | 0.267 | 0.2312 | No |
| 213 | ZNF850 |  |  | 589 | 0.266 | 0.2298 | No |
| 214 | ARHGEF39 |  |  | 595 | 0.264 | 0.2227 | No |
| 215 | TBC1D31 |  |  | 616 | 0.261 | 0.1871 | No |
| 216 | NDE1 |  |  | 623 | 0.259 | 0.1781 | No |
| 217 | CHCHD3 |  |  | 628 | 0.259 | 0.1728 | No |
| 218 | CENPI |  |  | 630 | 0.258 | 0.1733 | No |
| 219 | HNRNPUL1 |  |  | 637 | 0.257 | 0.1642 | No |
| 220 | NLGN4X |  |  | 641 | 0.257 | 0.1609 | No |
| 221 | CEP44 |  |  | 643 | 0.256 | 0.1613 | No |
| 222 | EXOSC8 |  |  | 647 | 0.256 | 0.1579 | No |
| 223 | UHRF1 |  |  | 652 | 0.255 | 0.1527 | No |
| 224 | DESI2 |  |  | 654 | 0.255 | 0.1531 | No |
| 225 | NUP37 |  |  | 655 | 0.255 | 0.1554 | No |
| 226 | RDX |  |  | 658 | 0.255 | 0.1540 | No |
| 227 | SPATA5 |  |  | 675 | 0.252 | 0.1258 | No |
| 228 | MYBL1 |  |  | 678 | 0.251 | 0.1243 | No |
| 229 | HMGCR |  |  | 683 | -0.257 | 0.1191 | No |
| 230 | GDI2 |  |  | 697 | -0.274 | 0.0968 | No |
| 231 | CHD7 |  |  | 706 | -0.284 | 0.0842 | No |
| 232 | ZEB1 |  |  | 717 | -0.317 | 0.0681 | No |
| 233 | TMEM256 |  |  | 724 | -0.345 | 0.0599 | No |
| 234 | HES6 |  |  | 752 | -0.523 | 0.0133 | No |
Table: GSEA details [plain text format]

  

Fig 2: RICHARDS\_NOWAKOWSKI\_SCIENCE\_2017\_MGE\_IPC1\_UPREG: Random ES distribution      
 Gene set null distribution of ES for **RICHARDS\_NOWAKOWSKI\_SCIENCE\_2017\_MGE\_IPC1\_UPREG**
